# A rapid, accessible real-time PCR approach to identify UBA1 somatic mutations in VEXAS syndrome

**DOI:** 10.3389/fmed.2026.1858546

**Published:** 2026-05-28

**Authors:** Luisa Agnello, Caterina Maria Gambino, Lidia La Barbera, Anna Masucci, Roberta Vassallo, Francesco Cacciabaudo, Mauro Midiri, Concetta Scazzone, Anna Maria Ciaccio, Giuliana Guggino, Marcello Ciaccio

**Affiliations:** 1 Department of Biomedicine, Neurosciences and Advanced Diagnostics, Institute of Clinical Biochemistry, Clinical Molecular Medicine, and Clinical Laboratory Medicine, University of Palermo, Palermo, Italy; 2 Department of Laboratory Medicine, University Hospital Paolo Giaccone, Palermo, Italy; 3 Department of Health Promotion, Mother and Child Care, Internal Medicine and Medical Specialties, Rheumatology Section, “P. Giaccone”, University of Palermo, Palermo, Italy; 4 Department of Health Promotion, Mother and Child Care, Internal Medicine and Medical Specialties, Institute of Legal Medicine, University of Palermo, Palermo, Italy; 5 Department of Health Promotion, Mother and Childcare, Internal Medicine and Medical Specialties, University of Palermo, Palermo, Italy

**Keywords:** allele-specific PCR, melting curve analysis, molecular diagnosis, real-time PCR, somatic mutations, UBA1, VEXAS syndrome

## Abstract

**Background:**

VEXAS (vacuoles, E1 enzyme, X-linked, autoinflammatory, somatic) syndrome is a severe adult-onset autoinflammatory disease caused by somatic mutations in the X-linked UBA1 gene, most commonly affecting codon 41. Early molecular confirmation is essential, but sequencing-based methods may be limited by turnaround time, cost, and sensitivity for low-level somatic variants. We aimed to validate a rapid, accessible allele-specific real-time PCR assay for detection of the most frequent UBA1 hotspot mutations associated with VEXAS syndrome.

**Methods:**

In this prospective monocentric study conducted at the University Hospital “P. Giaccone” (Palermo, Italy), 17 adults were enrolled: six patients with high clinical suspicion of VEXAS syndrome and eleven healthy controls. UBA1 variants c.121A>G, c.121A>C, and c.122T>C were screened using a SYBR Green–based allele-specific real-time PCR kit with mutation-specific reaction mixes and an internal housekeeping control, followed by melting curve analysis for variant discrimination. All PCR-positive samples were confirmed by Sanger sequencing.

**Results:**

Allele-specific real-time PCR identified UBA1 mutations in 5/6 (83.3%) suspected VEXAS cases. Sanger sequencing confirmed all real-time PCR–positive results, demonstrating 100% concordance between methods.

**Conclusion:**

This allele-specific real-time PCR assay enables rapid and reliable detection of the most common UBA1 codon 41 mutations associated with VEXAS syndrome using standard real-time PCR platforms. The approach provides a practical, cost-effective screening strategy to support timely diagnosis in patients with high clinical suspicion.

## 1 Introduction

VEXAS (Vacuoles, E1 enzyme, X-linked, Autoinflammatory, Somatic) syndrome is a recently identified adult-onset autoinflammatory disorder caused by somatic mutations in the X-linked UBA1 gene, which encodes the E1 ubiquitin-activating enzyme (1). It is estimated to affect nearly one million individuals worldwide, with pathogenic UBA1 variants present in approximately 1 in 13,600 people, although comprehensive global prevalence data remain limited. First described in 2020, VEXAS syndrome predominantly affects males over 50 years of age and presents with a complex constellation of rheumatologic, dermatologic, pulmonary, and hematologic manifestations, including recurrent fever, chondritis, neutrophilic dermatosis, pulmonary infiltrates, macrocytic anemia, and thrombocytopenia (1, 2). The syndrome is associated with significant morbidity and mortality, with reported mortality rates of 30–40%, making early and accurate diagnosis critical for appropriate clinical management (3, 4).

The molecular hallmark of VEXAS syndrome is the presence of somatic mutations in UBA1 gene, with more than 90% of cases harboring mutations at methionine 41 (p.Met41), specifically p. Met41Thr, p.Met41Val, and p.Met41Leu (5, 6). These mutations result in impaired ubiquitination, leading to dysregulated protein degradation, endoplasmic reticulum stress, and activation of innate immune pathways (7). The somatic nature of these mutations, occurring postzygotically in hematopoietic stem and progenitor cells, presents unique diagnostic challenges as the mutations are lineage-restricted, predominantly affecting myeloid cells while being absent in lymphoid lineages and fibroblasts (8). Genetic identification of VEXAS patients is essential for guiding appropriate management strategies and improving clinical outcomes.

Current diagnostic approaches for detecting UBA1 mutations include Sanger sequencing and next-generation sequencing (NGS) (4). While Sanger sequencing has been widely used and can detect mutations with variant allele fractions (VAFs) as low as 10–20%, it has limited sensitivity for detecting low-level somatic mutations (9, 10). NGS-based methods offer broader genomic coverage and can detect variants at VAFs ≥0.2 (corresponding to 20% variant allele frequency) with adequate sequencing depth (≥20×), but they are associated with longer turnaround time (TAT), higher costs, complex bioinformatic requirements, and may miss low-level mutations due to coverage limitations (11). Furthermore, the requirement for high sequencing depth and specialized analysis pipelines limits the accessibility of NGS testing in many clinical laboratories (12, 13).

Real-time PCR-based methods offer several advantages for detecting somatic mutations in clinical settings, including rapid TAT, high sensitivity, cost-effectiveness, and compatibility with standard molecular biology equipment available in most diagnostic laboratories (14–16). Allele-specific real-time PCR approaches can achieve detection sensitivities of 0.01–0.1% mutant allele fraction, significantly exceeding the capabilities of conventional sequencing methods (17–19). Recent studies have demonstrated the feasibility of developing targeted real-time PCR assays for detecting the most common UBA1 p. Met41 mutations, with high-resolution melting and allele-specific oligonucleotide PCR showing promise for both diagnostic screening and molecular monitoring of disease burden (13, 19).

Given the clinical urgency of diagnosing VEXAS syndrome, the predominance of recurrent hotspot mutations at p. Met41, and the limitations of current sequencing-based approaches, there is a critical need for rapid, sensitive, and widely accessible diagnostic methods. Real-time PCR-based detection of UBA1 mutations represents a practical solution that can facilitate earlier diagnosis, enable monitoring of VAFs during treatment, and improve access to genetic testing for patients with suspected VEXAS syndrome.

In this study, we describe the development and validation of a real-time PCR assay for the rapid and reliable detection of the three most common UBA1 somatic mutations associated with VEXAS syndrome.

## 2 Materials and methods

### 2.1 Study population

This observational, prospective, monocentric cohort study was performed at the University Hospital “P. Giaccone” of Palermo, Italy. We enrolled 17 adult patients: six patients with a high clinical suspicion of VEXAS syndrome and eleven healthy controls.

Patients exhibiting clinical manifestations and laboratory findings consistent with a presumptive diagnosis of VEXAS syndrome were recruited from the Unit of Rheumatology of the University Hospital “Paolo Giaccone” in Palermo, Italy. The clinical suspicion for VEXAS syndrome arises in adults with a combination of treatment-refractory systemic inflammation, hematologic abnormalities, such as macrocytic anemia, thrombocytopenia, and/or monocytopenia, and characteristic vacuolization of myeloid and erythroid precursors on bone marrow examination (4, 20, 21).

Healthy controls were adult volunteers with no history of inflammatory, autoimmune, hematologic, or neoplastic diseases recruited at the Institute of Clinical Biochemistry, Clinical Molecular Medicine, and Clinical Laboratory Medicine, University of Palermo, Italy.

All participants provided written informed consent prior to inclusion in the study, which was conducted in accordance with the principles of the Declaration of Helsinki.

### 2.2 Genotyping UBA1 gene

Venous peripheral blood samples were collected in K3EDTA tubes. Genomic DNA was isolated from 200 μL of whole blood using the MagNA Pure system (Roche Diagnostics, Indianapolis, IN) with the MagNA Pure LC DNA Isolation Kit I, following the manufacturer’s protocol.

DNA quantity was assessed spectrophotometrically. The optimal concentration of genomic DNA was 200–240 ng for reaction (recommended 260/280 nm ratio between 1.8 and 2.1), according to the manufacturer. DNA quality was assessed to minimize variability in assay performance. DNA integrity was evaluated by electrophoretic profiling, while purity was determined using spectrophotometric ratios (A260/A280 and A260/A230).

Genotyping of UBA1 mutations was performed using an allele-specific PCR (AS-PCR) kit (INF-005, BioMol Laboratories Srl, Italy), enabling the detection of three somatic variants in the UBA1 gene: c.121A>G, c.121A>C, and c.122T>C. Amplification was carried out using SYBR Green–based real-time PCR on an automated platform (Bio-Rad CFX96 Dx, Bio-Rad Opus Dx, or Agilent AriaDx).

Each sample was analysed in parallel using three mutation-specific reaction mixes targeting UBA1 c.121A>G, c.121A>C, and c.122T>C variants, together with an internal housekeeping gene used as amplification control (Table 1). The limit of detection is ≥5 ng of genomic DNA, the limit of blank >40 Cq with a reproducibility of 99.9%, and specificity and sensitivity of 99.9 and 98%, respectively. No-template control was included to exclude peaks potentially generated by primer dimers.

**Table 1:** Interpretation criteria for allele-specific PCR–based detection of UBA1 variants.

Housekeeping	Mix 1 (c.121A>G)	Mix 2 (c.121A>C)	Mix 3 (c.122T>C)	Interpretation
Tm 76.5 ± 1 °C	Positive for Tm 79 ± 1 °C	Negative for Tm 84.4 ± 1 °C	Negative for Tm 85.4 ± 1 °C	Presence mutation 121A>G
Tm 76.5 ± 1 °C	Negative for Tm 79 ± 1 °C	Positive for Tm 84.4 ± 1 °C	Negative for Tm 85.4 ± 1 °C	Presence mutation 121A>C
Tm 76.5 ± 1 °C	Negative for Tm 79 ± 1 °C	Negative for Tm 84.4 ± 1 °C	Positive for Tm 85.4 ± 1 °C	Presence mutation 122T>C
Tm 76.5 ± 1 °C	Negative for Tm 79 ± 1 °C	Negative for Tm 84.4 ± 1 °C	Negative for Tm 85.4 ± 1 °C	Absence mutations

**Figure 1:**
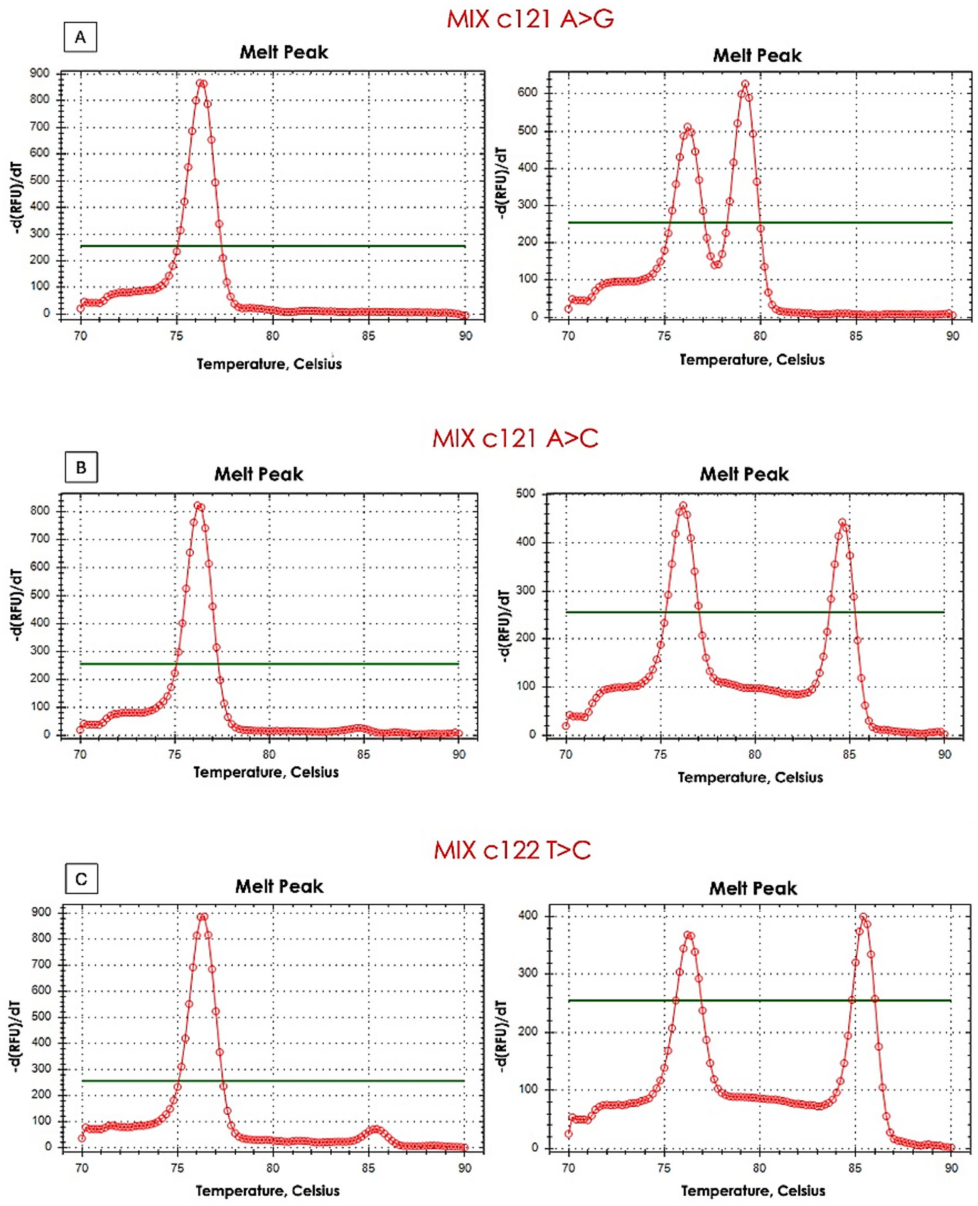
Representative melting curve analysis for allele-specific PCR detection of UBA1 variants. **(A)** UBA1 c.121A>G variant: absence (left) and presence (right) of the mutation. **(B)** UBA1 c.121A>C variant: absence (left) and presence (right) of the mutation. **(C)** UBA1 c.122T>C variant: absence (left) and presence (right) of the mutation.

Real-time PCR was performed under the thermal cycling conditions reported in Table 2, followed by a dissociation (melting curve) analysis from 70 °C to 90 °C with 0.2 °C increments. Fluorescence acquisition was carried out in the SYBR Green/FAM detection channel during amplification and melting curve analysis. Variant-specific amplification is identified based on characteristic melting temperature (Tm) peaks (79 ± 1 °C for the amplified c.121A>G UBA-1, 84.4 ± 1 °C for the amplified c.121A>C UBA-1, 85.4 ± 1 °C for the amplified c.122T>C UBA-1), while successful amplification of the housekeeping gene (76.5 ± 1 °C) was required to validate each run (Figure 1). Sequence annotation was based on the human reference genome GRCh38.p14 (chromosome X, NC_000023.11 - positions 47,198,799–47,199,299).

**Table 2:** Thermal cycling conditions and dissociation curve parameters for the real-time PCR assay.

Number of cycles	Temperature (°C)	Time
1 cycle	50 °C	2 min
1 cycle	94 °C	5 min
30 cycles	95 °C	50 s
	60 °C	40 s
	72 °C	50 s
1 cycle of dissociation	70 °C to 90 °C with an increase of 0.2 °C	

Serial dilution experiments were performed using wild-type and mutation-positive DNA samples mixed at different proportions to evaluate the analytical sensitivity and specificity of the assay.

Three synthetic constructs were synthesized, each comprising a 1,000-bp fragment that included the respective specific mutation, c.121A>G; c.121A>C; c.122T>C of UBA1 gene (Gene Universal, Newark DE 19713).

Three different mixes c.121A>G; c.121A>C and c.122T>C UBA-1 were evaluated against the three constructs showing amplification exclusively with the construct harboring its corresponding mutation, demonstrating complete absence of cross-reactivity. The assay was conducted in triplicate across three different real-time PCR platforms (Biorad Opus Dx, Agilent AriaDx e Thermofisher QuantStudio™ 5 Real-Time PCR System).

Assay sensitivity was assessed by using a construct containing the UBA-1 wild-type sequence (Gene Universal, Newark DE 19713) in combination with serial dilutions of each mutation-bearing construct. The assay was conducted in triplicate across three different real-time PCR platforms (Biorad Opus Dx, Agilent AriaDx e Thermofisher QuantStudio^™^ 5 Real-Time PCR System).

## 3 Results

**Figure 2:**
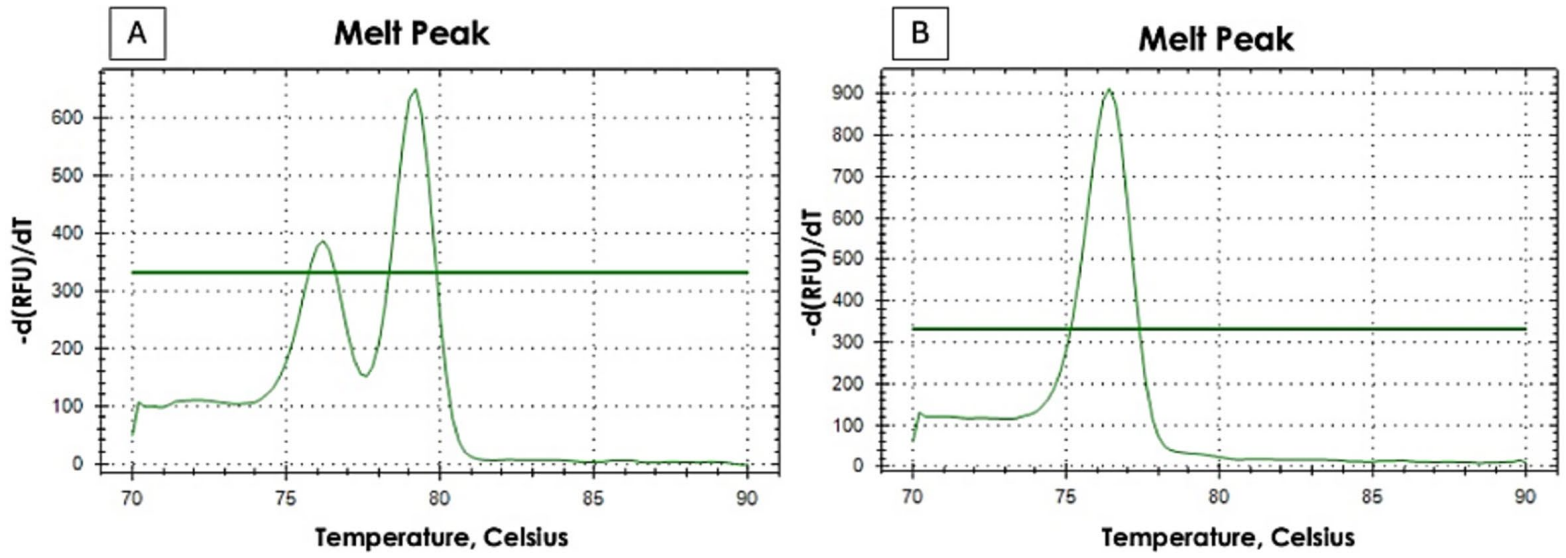
Melting curve analysis of a mutation-positive sample by allele-specific real-time PCR. **(A)** Positive melting curve profile; **(B)** Negative melting curve profile.

DNA analysis performed by allele-specific real-time PCR using BioRad platforms revealed the presence of UBA1 mutations in five of the six patients with suspected VEXAS syndrome. Specifically, four patients carried the c.121A>G variant, while one patient presented the c.121A>C variant. Representative melting curve profiles of mutation-positive and mutation-negative samples are shown in Figure 2.

**Figure 3:**
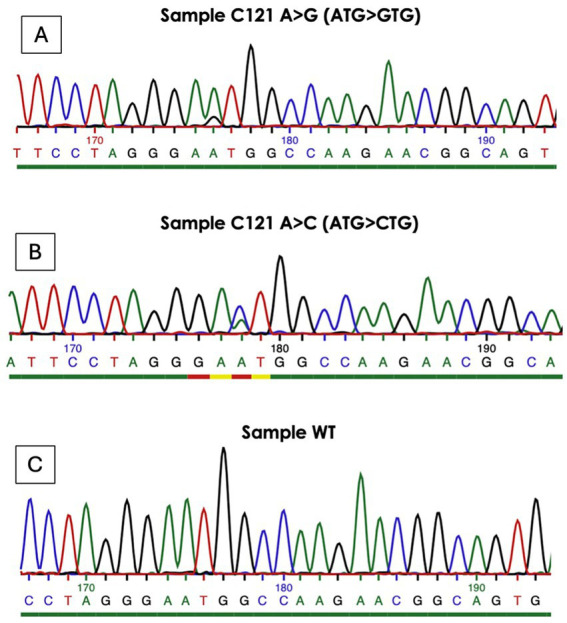
Sanger sequencing confirmation of UBA1 variants identified by real-time PCR. **(A)** Representative electropherogram of a sample harboring the c.121A>G (ATG>GTG) variant; **(B)** Representative electropherogram of a sample carrying the c.121A>C (ATG>CTG) variant; **(C)** Electropherogram of a wild-type (WT) control sample.

To confirm the real-time PCR findings, samples were further analysed by Sanger sequencing. Sequencing analysis confirmed the presence of the corresponding UBA1 variants in all real-time PCR–positive patients, validating the accuracy of the molecular screening results (Figure 3). The remaining sample was confirmed negative by real-time PCR.

To further evaluate assay specificity, the allele-specific real-time PCR method was tested on multiple cell lines known to be negative for the investigated UBA1 mutations. No mutation-specific amplification peaks were observed in any of the negative control cell lines, confirming the absence of non-specific amplification and supporting the analytical specificity of the assay. These findings further strengthen the reliability of the proposed method for routine molecular screening of UBA1 hotspot mutations associated with VEXAS syndrome (Table 3).

**Table 3:** Serial dilution experiments performed to evaluate the analytical sensitivity of the allele-specific real-time PCR assay for detection of UBA1 hotspot mutations associated with VEXAS syndrome.

Experiment	MIX PCR c.121A>G UBA1				MIX PCR c.121A>C UBA1				MIX PCR c.122T>C UBA1			
	UBA1 WT sequence	UBA1 c.121A>G mutated sequence	Mutated copies/(WT + mutated)	Amplification	UBA1 WT sequence	UBA1 c.121A>C mutated sequence	Mutated copies/(WT + mutated)	Amplification	UBA1 WT sequence	UBA1 c.122 T>C mutated sequence	Mutated copies/(WT + mutated)	Amplification
Experiment #1	5.4 × 10^6^ copies	5.4 × 10^6^ copies	50%	Present	5.4 × 10^6^ copies	5.4 × 10^6^ copies	50%	Present	5.4 × 10^6^ copies	5.4 × 10^6^ copies	50%	Present
Experiment #2	5.4 × 10^6^ copies	3.0 × 10^6^ copies	35.7%	Present	5.4 × 10^6^ copies	3.0 × 10^6^ copies	35.7%	Present	5.4 × 10^6^ copies	3.0 × 10^6^ copies	35.7%	Present
Experiment #3	5.4 × 10^6^ copies	1.5 × 10^6^ copies	25.4%	Present	5.4 × 10^6^ copies	1.5 × 10^5^ copies	25.4%	Present	5.4 × 10^6^ copies	1.5 × 10^6^ copies	25.4%	Present
Experiment #4	5.4 × 10^6^ copies	6 × 10^5^ copies	10%	Present	5.4 × 10^6^ copies	6 × 10^5^ copies	10%	Present	5.4 × 10^6^ copies	6 × 10^5^ copies	10%	Present
Experiment #5	5.4 × 10^6^ copies	3 × 10^5^ copies	5.26%	Present	5.4 × 10^6^ copies	3 × 10^5^ copies	5.26%	Present	5.4 × 10^6^ copies	3 × 10^5^ copies	5.26%	Present
Experiment #6	5.4 × 10^6^ copies	1.5 × 10^5^ copies	2.7%	Present	5.4 × 10^6^ copies	1.5 × 10^5^ copies	2.7%	Present	5.4 × 10^6^ copies	1.5 × 10^5^ copies	2.7%	Present
Experiment #7	5.4 × 10^6^ copies	0.75 × 10^5^ copies	1.36%	Present	5.4 × 10^6^ copies	0.75 × 10^5^ copies	1.36%	Present	5.4 × 10^6^ copies	0.75 × 10^5^ copies	1.36%	Present

Wild-type and mutation-positive DNA samples were mixed at decreasing mutant allele fractions for the three investigated variants (c.121A>G, c.121A>C, and c.122T>C). Amplification was successfully detected across all tested dilutions, including low mutant allele fractions down to 1.36%, supporting the analytical sensitivity and robustness of the assay.

## 4 Discussion

In this study, we developed and validated an allele-specific real-time PCR assay for the rapid detection of the three most prevalent UBA1 somatic mutations associated with VEXAS syndrome, namely c.121A>G, c.121A>C, and c.122T>C. Our findings demonstrate that this approach is reliable, sensitive, and readily applicable in a routine diagnostic laboratory setting, addressing several unmet needs in the current diagnostic pathway of this recently described and clinically severe disorder.

Allele-specific real-time PCR identified UBA1 mutations in five of six patients with suspected VEXAS syndrome. The remaining sample was negative by real-time PCR and was also confirmed as negative by Sanger sequencing. In addition, the assay was tested on multiple mutation-negative cell lines, confirming the absence of non-specific amplification. These findings support the clinical usefulness of real-time PCR as a rapid, cost-effective, and accessible diagnostic tool for VEXAS syndrome.

The high detection rate in our cohort aligns with current understanding of VEXAS syndrome epidemiology and diagnostic challenges. The American College of Rheumatology guidance statement emphasizes that genetic identification of UBA1 mutations is essential for definitive diagnosis and appropriate management of VEXAS syndrome (4). Our real-time PCR approach specifically targets the three most prevalent mutations at codon 41 (c.121A>G, c.121A>C, and c.122T>C), which account for more than 90% of reported VEXAS cases (4). This targeted approach provides a practical balance between comprehensive coverage and diagnostic efficiency. The advantages of real-time PCR-based detection over conventional sequencing methods are particularly relevant for VEXAS syndrome diagnosis. While Sanger sequencing can identify mutations with VAFs roughly above 20%, it lacks sensitivity to detect lower mutational burdens that may still cause clinically significant disease (4, 10). In contrast, allele-specific PCR methods can achieve detection sensitivities ranging from 0.01 to 0.1% mutant allele fraction, significantly exceeding conventional sequencing capabilities (22–24). This enhanced sensitivity is critical given that recent population-based studies have identified VEXAS-associated UBA1 variants with VAFs as low as 4.5%, and disease severity appears to correlate with VAF levels (25, 26).

The melting curve analysis employed in our assay provides a straightforward readout that can be interpreted in real-time without requiring complex bioinformatic analysis. High-resolution melting analysis has demonstrated high sensitivity (97.5% overall) for detecting disease-associated mutations in humans, with even higher sensitivity (98.7%) when using modern instruments (27). The technique is particularly well-suited for detecting known hotspot mutations, as in VEXAS syndrome, where the majority of pathogenic variants cluster at specific codons (28, 29). The characteristic Tm peaks for each mutation (79 ± 1 °C for c.121A>G, 84.4 ± 1 °C for c.121A>C, and 85.4 ± 1 °C for c.122T>C) provide clear discrimination between wild-type and mutant alleles, with the housekeeping gene control (76.5 ± 1 °C) ensuring assay validity.

The clinical implications of rapid UBA1 mutation detection are substantial. VEXAS syndrome is associated with significant morbidity and mortality rates of 30–40%, making early and accurate diagnosis critical for appropriate clinical management (4). The syndrome presents diagnostic challenges due to its overlap with other rheumatologic and hematologic conditions, including relapsing polychondritis, Still’s disease, and myelodysplastic syndromes (1). Real-time PCR can facilitate earlier diagnosis by providing results within hours rather than the days to weeks typically required for NGS or even standard Sanger sequencing, potentially enabling more timely therapeutic intervention.

Our assay design incorporates several technical features that enhance its robustness and clinical applicability. The use of SYBR Green-based detection eliminates the need for expensive labelled probes, reducing costs while maintaining high specificity (30, 31). The parallel analysis of three mutation-specific reaction mixes with an internal housekeeping control ensures comprehensive screening while minimizing false-negative results. This approach is consistent with established principles for allele-specific PCR design, where the introduction of artificial mismatches and appropriate primer design can significantly enhance discrimination between wild-type and mutant alleles (32, 33).

The compatibility of our assay with multiple real-time PCR platforms (Bio-Rad CFX96 Dx, Bio-Rad Opus Dx, and Agilent AriaDx) enhances its accessibility for clinical laboratories. This platform flexibility is particularly important given that real-time PCR instruments are widely available in diagnostic laboratories, unlike the specialized equipment and bioinformatic infrastructure required for NGS (34). The relatively modest DNA input requirement (200–240 ng per reaction) and straightforward workflow make the assay suitable for routine clinical implementation. However, several limitations of our approach warrant consideration. First, the assay specifically targets only the three most common mutations at codon 41 and does not detect mutations at other positions, such as p.Ser56, which are associated with milder inflammatory phenotypes of VEXAS (4). Recent reports have also identified pathogenic mutations outside of exon 3 that can cause VEXAS-like clinical presentations (4). Therefore, while our assay provides rapid screening for the most common mutations, negative results in patients with high clinical suspicion should prompt more comprehensive genetic testing using NGS or whole-exome sequencing to ensure complete coverage of the UBA1 gene. Second, while allele-specific PCR can achieve very high analytical sensitivity, the clinical sensitivity depends on the VAF in the sample tested. UBA1 mutations in VEXAS syndrome are lineage-restricted, predominantly affecting myeloid cells while being largely absent in lymphoid lineages. The detection of mutations in peripheral blood may be compromised by certain treatments, particularly hypomethylating agents such as azacitidine, which can reduce VAFs. In cases where peripheral blood testing is negative despite high clinical suspicion, bone marrow-derived samples may yield higher VAFs and improved detection rates (4). Third, our study cohort was relatively small (*n* = 17, with 6 suspected VEXAS cases), limiting our ability to comprehensively assess assay performance characteristics across a broad range of VAFs and clinical presentations. Larger validation studies are needed to establish definitive sensitivity and specificity metrics, determine the lower limit of detection in clinical samples, and evaluate performance across diverse patient populations. Additionally, longitudinal studies would be valuable to assess the utility of real-time PCR for monitoring VAF changes during treatment, as recent evidence suggests that VAF levels may correlate with disease activity and treatment response (10).

The cost-effectiveness of real-time PCR compared to NGS is another important consideration for clinical implementation. While NGS provides comprehensive genomic coverage and can detect variants at multiple loci simultaneously, it is associated with higher costs, longer TAT, and complex bioinformatic requirements (7). For targeted detection of known hotspot mutations, real-time PCR offers a more economical alternative that can be implemented in laboratories with standard molecular biology equipment. This is particularly relevant for resource-limited settings or for initial screening in patients with suspected VEXAS syndrome, with NGS reserved for cases requiring more comprehensive analysis. Future directions for this work include expanding the assay to cover additional UBA1 mutations, including those at p. Ser56 and splice site variants such as c.118-1G>C, which together account for a significant proportion of VEXAS cases. Development of multiplex assays that can simultaneously detect multiple mutations in a single reaction would further enhance efficiency and reduce costs. Additionally, integration of quantitative analysis capabilities would enable precise VAF determination, which has prognostic implications and could guide treatment decisions.

In conclusion, our allele-specific real-time PCR assay provides a rapid, sensitive, and cost-effective method for detecting the most common UBA1 mutations associated with VEXAS syndrome. The assay demonstrated 100% concordance with Sanger sequencing in our validation cohort and offers several practical advantages for clinical implementation, including short TAT, compatibility with standard laboratory equipment, and straightforward interpretation. While the assay has limitations in terms of mutation coverage and requires confirmation of negative results in high-suspicion cases, it represents a valuable addition to the diagnostic toolkit for VEXAS syndrome. As awareness of this recently described condition grows and the number of patients requiring genetic testing increases, accessible and efficient diagnostic methods will be crucial for ensuring timely diagnosis and appropriate management of this serious and potentially life-threatening disorder (35).

## Data Availability

The raw data supporting the conclusions of this article will be made available by the authors, without undue reservation.

## References

[1] Corrao S Moschetti M Scibetta S Calvo L Giardina A Cangemi I . VEXAS syndrome: genetics, gender differences, clinical insights, diagnostic pitfalls, and emerging therapies. Int J Mol Sci. (2025) 26:7931. doi: 10.3390/ijms26167931, 40869252 PMC12386336

[2] Bindoli S Morello-Pasin G Guidea I Padoan R Iorio L Bixio R . VEXAS syndrome in rheumatology practice: features from a multicenter cohort in north-East Italy. Front Immunol. (2025) 16:1700737. doi: 10.3389/fimmu.2025.1700737, 41409287 PMC12705374

[3] Holden R Jeelal Y McLean-Tooke A Pathmanathan K Nolan D. VEXAS: a review of current understandings and emerging treatment strategies. Front Immunol. (2025) 16:1644404. doi: 10.3389/fimmu.2025.1644404, 40791602 PMC12336205

[4] Mekinian A Georgin-Lavaille S Ferrada MA Savic S Koster MJ Kosmider O . American College of Rheumatology guidance statement for diagnosis and management of VEXAS. Arthritis Rheumatol. (2025) 78:509–22. doi: 10.1002/art.43287 40787890 PMC12991921

[5] Ferrada MA Savic S Cardona DO Collins JC Alessi H Gutierrez-Rodrigues F . Translation of cytoplasmic UBA1 contributes to VEXAS syndrome pathogenesis. Blood. (2022) 140:1496–506. doi: 10.1182/blood.2022016985, 35793467 PMC9523373

[6] Hernández-Rodríguez J Mensa-Vilaró A Aróstegui JI. Paradigm shift in monogenic autoinflammatory diseases and systemic vasculitis: the VEXAS syndrome. Med Clin (Barc). (2022) 159:489–96. doi: 10.1016/j.medcli.2022.06.018, 36049972

[7] Narendra VK Das T Wierciszewski LJ Londoner RJ Morrison JK Martindale P . Independent mechanisms of inflammation and myeloid bias in VEXAS syndrome. Nature. (2025) 649:1273–81. doi: 10.1038/s41586-025-09815-0 41183570 PMC12851934

[8] Beck DB Bodian DL Shah V Mirshahi UL Kim J Ding Y . Estimated prevalence and clinical manifestations of UBA1 variants associated with VEXAS syndrome in a clinical population. JAMA. (2023) 329:318–24. doi: 10.1001/jama.2022.24836, 36692560 PMC10408261

[9] Maeda A Tsuchida N Uchiyama Y Horita N Kobayashi S Kishimoto M . Efficient detection of somatic UBA1 variants and clinical scoring system predicting patients with variants in VEXAS syndrome. Rheumatology. (2024) 63:2056–64. doi: 10.1093/rheumatology/kead425, 37606963

[10] von Bornemann Fløe L Dyrmose KO Sørensen CD Nørgaard M Pedersen FKU Vad-Nielsen J . Diagnostic and monitoring strategies for VEXAS syndrome: evaluating sanger sequencing, NGS, and the SWIM-score. J Clin Immunol. (2025) 45:138. doi: 10.1007/s10875-025-01932-9, 41026267 PMC12484315

[11] Koster MJ Lasho TL Olteanu H Reichard KK Mangaonkar A Warrington KJ . VEXAS syndrome: clinical and hematologic features and a practical approach to diagnosis and management. Am J Hematol. (2024) 99:284–99. doi: 10.1002/ajh.27156 37950858

[12] Hagiya A Siddiqi IN Wang E Lu CM. How I diagnose and manage VEXAS syndrome. Am J Clin Pathol. (2024) 162:28–40. doi: 10.1093/ajcp/aqae017, 38511841

[13] Martín Castillo I Mora E Hernani R Cervera JV Fernandez MJ Ferrer-Lores B . Rapid screening and monitoring of UBA1 mutations in VEXAS syndrome. J Mol Diagn. (2025) 27:431–7. doi: 10.1016/j.jmoldx.2025.03.004, 40239805

[14] Morlan J Baker J Sinicropi D. Mutation detection by real-time PCR: a simple, robust and highly selective method. PLoS One. (2009) 4:e4584. doi: 10.1371/journal.pone.0004584, 19240792 PMC2642996

[15] Vargas DY Kramer FR Tyagi S Marras SA. Multiplex real-time PCR assays that measure the abundance of extremely rare mutations associated with cancer. PLoS One. (2016) 11:e0156546. doi: 10.1371/journal.pone.0156546, 27244445 PMC4887114

[16] Wang H Jiang J Mostert B Sieuwerts A Martens JW Sleijfer S . Allele-specific non-extendable primer blocker PCR for DNA mutation detection in cancer. J Mol Diagn. (2013) 15:62–9. doi: 10.1016/j.jmoldx.2012.08.007, 23159590

[17] Kreutzinger V Pankow A Boyadzhieva Z Schneider U Ziegeler K Stephan LU . VEXAS and myelodysplastic syndrome: an interdisciplinary challenge. J Clin Med. (2024) 13:1049. doi: 10.3390/jcm13041049, 38398362 PMC10889042

[18] Li J Wang L Jänne PA Makrigiorgos GM. Coamplification at lower denaturation temperature PCR increases mutation-detection selectivity of TaqMan-based real-time PCR. Clin Chem. (2009) 55:748–56. doi: 10.1373/clinchem.2008.113381, 19233916 PMC2754313

[19] Wadsworth PA Chen SB Lawrence L Ho CC Le JE Libiran P . Rapid clinical deployment of UBA1 testing in patients with VEXAS syndrome. Am J Clin Pathol. (2025) 164:360–6. doi: 10.1093/ajcp/aqaf051, 40418703 PMC13016694

[20] Lim Y Park IH Lee HH Baek K Lee BC Cho G. Modified Taq DNA polymerase for allele-specific ultra-sensitive detection of genetic variants. J Mol Diagn. (2022) 24:1128–42. doi: 10.1016/j.jmoldx.2022.08.002, 36058471 PMC9746316

[21] Markou A Tzanikou E Ladas I Makrigiorgos GM Lianidou E. Nuclease-assisted minor allele enrichment using overlapping probes-assisted amplification-refractory mutation system. Anal Chem. (2019) 91:13105–11. doi: 10.1021/acs.analchem.9b03325, 31538770 PMC7154944

[22] Anderson M Ercelen D Richardson A Kim J Torene RI Sirenko M . Clinical manifestations of VEXAS syndrome across a broad spectrum of UBA1 mutation burden. Arthritis Rheumatol. (2025) 78:223–30. doi: 10.1002/art.43327, 40976851 PMC12906865

[23] Corty RW Brogan J Byram K Springer J Grayson PC Bick AG. VEXAS-defining UBA1 somatic variants in 245,368 individuals. Arthritis Rheumatol. (2024) 76:942–8. doi: 10.1002/art.42802, 38225170 PMC11410361

[24] Li BS Wang XY Ma FL Jiang B Song XX Xu AG. Accuracy of high-resolution melting analysis for detection of disease-associated mutations: a meta-analysis. PLoS One. (2011) 6:e28078. doi: 10.1371/journal.pone.0028078 22194806 PMC3237421

[25] Mehrotra M Patel KP. High-resolution melt curve analysis in cancer mutation screening. Methods Mol Biol. (2016) 1392:63–9. doi: 10.1007/978-1-4939-3360-0_7, 26843047

[26] Wittwer CT Hemmert AC Kent JO Rejali NA. DNA melting analysis. Mol Asp Med. (2024) 97:101268. doi: 10.1016/j.mam.2024.101268, 38489863

[27] Bruno A Gurnari C Alexander T Snowden JA Greco R. Autoimmune manifestations in VEXAS: opportunities for integration and pitfalls to interpretation. J Allergy Clin Immunol. (2023) 151:1204–14. doi: 10.1016/j.jaci.2023.02.017, 36948992

[28] van der Velden VH Hochhaus A Cazzaniga G Szczepanski T Gabert J van Dongen JJ. Detection of minimal residual disease in hematologic malignancies by real-time quantitative PCR. Leukemia. (2003) 17:1013–34. doi: 10.1038/sj.leu.2402922 12764363

[29] Yang Z Zhao N Chen D. Improved detection of BRAF V600E using allele-specific PCR coupled with controllers. Sci Rep. (2017) 7:13817. doi: 10.1038/s41598-017-14140-2 29061997 PMC5653796

[30] Lefever S Rihani A Van der Meulen J Pattyn F Van Maerken T Van Dorpe J . Cost-effective and robust genotyping using double-mismatch allele-specific qPCR. Sci Rep. (2019) 9:2150. doi: 10.1038/s41598-019-38581-z, 30770838 PMC6377641

[31] Shimony S Stahl M Stone RM. Acute myeloid leukemia: 2025 update on diagnosis, risk stratification, and management. Am J Hematol. (2025) 100:860–91. doi: 10.1002/ajh.27625, 39936576 PMC11966364

[32] Lopez A Patel S Geyer JT Racchumi J Chadburn A Simonson P . Comparison of multiple clinical testing modalities for assessment of NPM1-mutant AML. Front Oncol. (2021) 11:701318. doi: 10.3389/fonc.2021.701318, 34527579 PMC8435844

[33] Sepulveda AR Hamilton SR Allegra CJ Grody W Cushman-Vokoun AM Funkhouser WK . Molecular biomarkers for the evaluation of colorectal cancer. J Clin Oncol. (2017) 35:1453–86. doi: 10.1200/JCO.2016.71.9807, 28165299

[34] Groarke EM Turturice B Patel BA Quinn KA Fike A Grayson PC. VEXAS syndrome: pathogenesis, clinical spectrum, and therapeutic strategies. Lancet. (2026) 407:637–48. doi: 10.1016/S0140-6736(25)02164-6 41520673

[35] Al-Hakim A Goldberg S Gaillard S Heiblig M Beck DB Savic S. Clinical features in VEXAS syndrome: a systematic review. Rheumatology. (2025) 64:5217–29. doi: 10.1093/rheumatology/keaf293, 40570089 PMC12494223

